# Health Needs of Adults with Intellectual Disability Relevant for the Family Physician

**DOI:** 10.1100/tsw.2003.91

**Published:** 2003-10-05

**Authors:** Joav Merrick, Isack Kandel, Mohammed Morad

**Affiliations:** National Institute of Child Health and Human Development, Office of the Medical Director, Division for Mental Retardation, Ministry of Social Affairs, Box 1260, IL-91012, USA

**Keywords:** developmental disability, mental retardation, intellectual disability, aging, health concerns, public health, Israel

## Abstract

People with developmental disability, mental retardation, or intellectual disability are living longer and becoming prone to age-related health problems and diseases of old age much like the general population. This worldwide trend is also seen in Israel, where today 39.8% of persons with intellectual disability in residential care are 40 years old and above. There is a need for service and staff providers to receive training; a need for more research and better service for this aging population. This review presents health concerns for older persons with different levels of intellectual disability, health concerns in persons with Down syndrome, and persons with epileptic seizures and cerebral palsy in relation to general practice and family medicine. The review is concluded with recommendations on health and aging in adults with intellectual disabilities and the call for formalized training in the topic for specialists in family medicine.

## INTRODUCTION

Increased life expectancy for persons with intellectual disability (ID), which now is coming close to that of the general population, has resulted in a larger population of older persons with ID worldwide. One of the exceptions is the group with Down syndrome (DS), where the life expectancy today is 56 years or about 20 years below that of the general population.

In Israel (total population of over 6 million), the Division for Mental Retardation (DMR) is in contact with approximately 23,000 persons of all ages. Residential care is provided to about 6,000 persons in close to 60 residential care centers countrywide. In addition, 2,000 persons receive residential care in hostels or protected apartments within the community. More than 50 other settings provide vocational and educational services to 15,000 persons in day-care kindergartens, day-treatment centers, sheltered workshops, or integrated care within the community.

The age distribution of 6,370 persons with ID in residential care centers from 2001 can be seen in Table 1[1], while the level of ID in residential care centers in Israel is seen in Table 2[1]. In 2001, there were 9 government-operated residential care centers, 33 privately owned, and 12 public with a mean of 117.96 persons in each center (range 25–398). It is worthy to note that the total expenditure is provided by the government, which also operates the government institutions. Private and public institutions have their own independent administration, but receive funding, clients, and supervision by the government. The total expenditure of the DMR today is close to 200 million dollars per annum (for trends see[2]). The profile of the population in residential care centers is shown in Table 3. In Table 4, both community and residential care service is compared for the years 1990 and 2000. Here an increase in the number of persons served can be seen as well as new services implemented over that period. In Table 5, the number of persons in residential care can be seen together with the number of deaths per year and mortality rate[3].

**TABLE 1**
**Population of Persons with ID in Residential Care Centers (Institutions) in Israel (2001)[1]**

| Age (Years) | Males | Females | Total | Percent |
|---|---|---|---|---|
| 0–9 | 101 | 86 | 187 | 2.93 |
| 10–19 | 502 | 379 | 881 | 13.83 |
| 20–39 | 1,635 | 1,130 | 2,765 | 43.41 |
| 40–49 | 732 | 652 | 1,384 | 21.73 |
| 50–59 | 419 | 423 | 842 | 13.22 |
| >60 | 147 | 164 | 311 | 4.88 |
| **Total** | **3,536** | **2,834** | **6,370** | **100.00** |
| **Percent** | **55.51** | **44.49** | **100.00** | |

**TABLE 2**
**Level of ID in the Population of Persons with ID in Residential Care Centers in Israel (2001)**

| Age (Years) | Mild | Moderate | Severe | Profound | Other | Total | Percent |
|---|---|---|---|---|---|---|---|
| 0–9 | 2 | 13 | 105 | 67 | 0 | 187 | 2.93 |
| 10–19 | 36 | 299 | 351 | 195 | 0 | 881 | 13.83 |
| 20–39 | 217 | 985 | 954 | 601 | 8 | 2,765 | 43.41 |
| 40–49 | 150 | 641 | 438 | 152 | 2 | 1,384 | 21.73 |
| 50–59 | 81 | 440 | 237 | 79 | 5 | 842 | 13.22 |
| >60 | 27 | 161 | 93 | 27 | 3 | 311 | 4.88 |
| **Total** | **513** | **2,540** | **2,178** | **1,121** | **18** | **6,370** | **100.00** |
| **Percent** | **8.05** | **39.88** | **34.19** | **17.60** | **0.28** | **100.00** | |

Mild ID: IQ 55–70, moderate: IQ 35–54, severe: IQ 20–34, and profound: IQ < 20. Other are 18 persons, who historically were placed in institutions for other reasons and preferred to stay on, since they regarded the institution as their home[1].

**TABLE 3**
**Profile of the Population in Residential Care Centers in Israel (2001)[1]**

| Profile | Numbers | Percent |
|---|---|---|
| Educational | 727 | 11.41 |
| Treatment | 1,370 | 21.51 |
| Rehabilitation | 511 | 8.02 |
| Nursing | 1,578 | 24.77 |
| Challenging behavior | 2,184 | 34.29 |
| **Total** | **6,370** | **100.00** |

*Educational* is the term for persons with a level of self-care, social skills, and ability to work. *Treatment* refers to persons with normal to moderate self-care functions and social skills, able to accomplish simple tasks and skills in a limited number of activities. *Rehabilitation* refers to persons with poor adaptability skills, in need of support, instruction, and assistance. *Nursing* refers to persons in constant need of supervision, care, and who are dependent on the environment. *Challenging behavior* refers to persons that are hyperactive and in need of constant supervision.

**TABLE 4**
**Comparison between both Residential Care and Community Services for People with ID in Israel (1990–2000)**

| Type of Service | 1990 | 2000 |
|---|---|---|
| Assessment centers<br>Total assessments | 1,000 | 2,000 |
| Kindergartens<br>For nursing and treatment | 900 | 1,920 |
| Day-care for mild ID | 1,600 | 1,300 |
| After hour care | 700 | 2,400 |
| Leisure care | 1,400 | 1,750 |
| Home help assistance | 150 | 250 |
| Sheltered workshops | 1,900 | 2,550 |
| Long-day care | 0 | 665 |
| Integrated care in community (age 0–3 years) | 0 | 140 |
| TOTAL Community | 7,650 | 12,975 |
| TOTAL Residential | 5,376 | 7,819 |
| TOTAL Service DMR | 13,026 | 20,794 |

With more persons with ID living longer comes an increase in age-related health problems such as seen in the general population including heart disease, cardiovascular disease, cancer, visual and hearing impairment. Since many physicians and care staff involved with the health service of persons with ID come from the field of pediatrics, there will be a need to take a long-term view and also involve the fields of family medicine and geriatrics in the care. The physician working in family medicine in the community in Israel has very little training or experience working with ID, in spite of more people with ID in Israel living today in the community compared to residential care (17,000 vs. 6,000).

**TABLE 5**
**Number of Persons with ID in Residential Care Centers and Yearly Mortality and Mortality Rate in Israel during 1991–97[3]**

| Institution | 1991 | 1992 | 1993 | 1994 | 1995 | 1996 | 1997 |
|---|---|---|---|---|---|---|---|
| Government | 2.252 | 2.230 | 2.224 | 2.226 | 2.158 | 2.107 | 2.080 |
| Public | 1.166 | 1.166 | 1.303 | 1.422 | 1.489 | 1.550 | 1.650 |
| Private | 1.952 | 1.952 | 2.242 | 2.401 | 2.738 | 2.900 | 3.250 |
| Foster family | 245 | 245 | 235 | 240 | 240 | 240 | 240 |
| LTC Hospital | 10 | 10 | 0 | 15 | 15 | 11 | 10 |
| **TOTAL** | **5.625** | **5.603** | **6.004** | **6.304** | **6.640** | **6.808** | **7.230** |
| | | | | | | | |
| Number of deaths | 59 | 70 | 65 | 60 | 60 | 64 | 72 |
| Mortality rate (per 1,000) | 10.49 | 12.49 | 10.83 | 9.52 | 9.04 | 9.40 | 9.96 |

In this paper we would like to share some of our experiences of health-related problems and the needs of adult persons with ID relevant for family physicians working with persons with ID both in residential care or in the community.

## HEALTH CONCERNS IN PERSONS WITH MILD AND MODERATE INTELLECTUAL DISABILITY

The concerns will be the same as for the general aging population, but due to communication problems, lack of service, sometimes lack of interest, or commitment from the service providers, diagnosis or treatment can be ignored, overlooked, or delayed for this population[4]. This is unfortunate, because simple medical problems that could be solved will have adverse effects on the quality of life for the person with ID.

Cardiovascular disease was found to be less in persons with ID aged 65 years and older admitted to nursing homes than persons without ID (24.4 vs. 56.5%)[5], but there are very few studies on the prevalence of heart disease in this population. A recent study from New York State[6] of 1,371 adult persons with ID living in the community showed that cardiovascular disease increased with increasing age and was more likely among adults who were more functional, than in adults with seizures and with the highest BMI.

The incidence of death from cancer in the U.K. for persons with ID has been lower than the general population (11.7–17.5 vs. 26%), but the incidence of cancer is now on the increase due to increased longevity[7,8]. In New York State, cancer increased significantly with age and was more likely to occur in females[6]. Gastrointestinal cancer was proportionally higher than the general population (48–58.5 vs. 25% of cancer deaths in the U.K.)[7,8]. In a study of cancer and DS, a statistically significant excess of leukemia was found and, in addition, an excess of gastric cancer in institutionalized males was observed[9].

Women with ID are much less likely to undergo cervical smear tests than the general population (19 vs. 77%)[10] and also less likely to have breast cancer examinations or receive mammography[11].

Visual and hearing impairment affect about half of the general population over 65 years of age and will also be seen in older persons with ID. Visual impairment was found seven times higher in adults with ID than the general population[12]. Cataract, glaucoma, macular degeneration, and diabetic retinopathy should be considered. Ocular and orbital abnormalities in persons with DS are numerous and reported with various occurrence: blepharitis (2–47%), keratoconus (5–8%), glaucoma (less than 1%), iris anomalies (38–90%), cataract (25–85%), retinal anomalies (0–38%), optic nerve anomalies (very few cases), strabismus (23–44%), amblyopia (10–26%), nystagmus (5–30%), and refractive errors (18–58%)[13]. Deafness is also common in this population[14] and impairment increases with increasing age[6,14]. Presbycusis (high pitched tones become harder to hear) and hearing loss is also seen in this population as a result of impacted earwax[15].

Constipation is seen frequently with this population and can be a lifelong problem, which increases with age due to decline in mobility and less bowel motility or movement. Medication can also be a factor in constipation. Caretakers need to be aware of this problem, because of the dangers of accumulation and sometimes even intestinal perforation and death[16].

With aging, the bladder capacity and muscle tone will decrease and cause urinary incontinence and in men enlargement of the prostate gland can also restrict urinary flow.

Dental problems in surveys of this population have identified poor oral hygiene, high prevalence of gingival disease, and untreated dental caries with dental care difficult to implement[17].

## HEALTH CONCERNS IN PERSONS WITH SEVERE AND PROFOUND INTELLECTUAL DISABILITY

Many persons with severe and profound ID will have associated medical problems and disease. In a recent review of mortality in this population in Israel[3] for the 1991–97 period, 60% of the 450 cases were deaths before the age of 41 years and 68% in the severe-profound group. Cardiovascular reasons accounted for 35%, respiratory disease for 25%, and infectious diseases for 9% of the cases. The group with severe and profound ID, who survive into old age, will therefore have musculoskeletal problems, respiratory disease, problems with swallowing, some will need gastrostomy and therefore demand a high service level.

Osteoporosis and increased fractures should be kept in mind in this population[18] due to immobility, nutrition, and lack of activity.

## HEALTH CONCERNS IN DOWN SYNDROME

In the 1920s, the life expectancy for persons with DS was only 9 years, but this has now increased to 56 years, which is still 20 years less than the general population[19].

Alzheimer's disease (AD) was first described by professor Alois Alzheimer, Germany, in 1906[20], when he reported the case of Auguste D, a 51-year-old female patient, whom he had followed at a Frankfurt hospital since 1901 up until her death on April 8th, 1906. Even after her death, he went on to study the neuropathological features of her illness. Shortly after her death, he presented her case at the 37th Conference of German Psychiatrist in Tubingen on November 4th, 1906 in which he described her symptoms:

- Progressive cognitive impairment
- Focal symptoms
- Hallucinations
- Delusions
- Psychosocial incompetence
- Neurobiological changes found at autopsy: plaques, neurofibrillary tangles, and artheriosclerotic changes

These symptoms are still the characteristics of AD today, which is the most common cause of dementia in western countries. Clinically, AD most often presents with a subtle onset of memory loss followed by a slowly progressive dementia that has a course of several years. The duration of AD can be 3–10 years from diagnosis to death and this progress is more rapid in persons with DS. Usually seizures will present at the end stage. Epilepsy is seen in about 5% of persons with DS, but the combination of DS and AD will produce epileptic seizures in 85% of the cases.

In addition, AD persons with DS are prone to other medical problems when aging. In a recent study from Holland[21], 96 adults with DS from an institution were investigated systematically over the period 1991–95 with cytogenetic diagnosis, mental functioning, dementia, ophthalmological and audiological assessments, and thyriod function. Of this group, 73% were older than 40 years and only 4.3 % were females; 3% had mild ID, 82% moderate and severe ID, and 15% profound ID; 19% had already dementia, but this number increased to 42% in persons above 50 years of age.

Epilepsia was present in 16.7% of all the persons, but in 50% of those with dementia. Vision problems were frequent with only 17% with normal vision and here again can be seen an increase of problems with an increase in age. In the 50–59 year age group, 44.8% had moderate to severe vision loss; 70% had moderate, severe, or very severe hearing loss, which was undiagnosed before systematic hearing tests were performed; 49% had thyroid dysfunction.

In addition to the above-observed health problems, persons with DS have increased obesity, premature aging of the immune system resulting in various diseases, increased sleep apnea, and musculoskeletal problems[22].

## EPILEPSY AND CEREBRAL PALSY

Persons with ID, epilepsy, and seizures, who receive antiepileptic drugs over long periods, will also be at an increased risk for premature mortality, increased risk of osteoporosis, and increased risks of falls.

A study of risk factors for injuries and falls[23] among 268 adults with ID from 18 nursing homes in Chicago revealed that 11% (30 cases) had reported injuries 12 months prior to the follow-up study. Over 50% of the injuries were caused by falls and persons with a higher frequency of seizures, more destructive behavior, and usage of antipsychotic drugs had the highest risk of injuries. Further analysis of the data revealed that persons over 70 years of age, ambulatory, and with a higher frequency of seizures had the highest risk of injurious falls.

Studies of the effect of aging in persons with cerebral palsy (CP) are few and studies with persons with both ID and CP are even less. Reason would tell you that mobility would be decreased over the years, osteoporosis increased, and dependency on care increased over the years.

## INTELLECTUAL DISABILITY AND GENERAL PRACTICE

The number of persons with ID in general practice has been studied in Holland[24]. A general practice database with 62,000 patients had 318 persons (or 0.65%) with ID, which together with persons in residential case showed a total prevalence of 0.82%.

Barriers to treatment in general practice have been studied in Australia among 912 randomly selected general practitioners (GP)[25], where communication difficulties with patients and other health professionals, and problems in getting patient histories were the most significant barriers. Other difficulties were lack of training and experience, poor patient compliance, consultation time limitations, difficulties in problem determination, examination difficulties, poor continuity of care, and inadequate knowledge of services and resources.

Researchers in Cardiff, Wales have developed a health screening tool for persons with ID in general practice[26], which has also been used in general practice in New Zealand[27]. Here the introduction of an annual health screen resulted in medical findings of 72.6% of the 1,311 persons screened, which afterward required follow-up interventions.

A survey conducted through all U.S. family practice residency directors[28] showed that 84% of the programs had provided residents with one or more experience about health care needs of persons with ID and 60% had instructed residents in that area.

In the year 2000, Holland established a chair in intellectual disability at the University of Rotterdam Department of Family Medicine and started a 3-year subspecialty in ID[29], where the first class of specialists in ID will graduate in November 2003.

## CONCLUSION

We have seen an increase in the population of persons with ID surviving into older age over recent years resulting in health problems emerging as in the general population, but sometimes at a much earlier stage, like AD and DS beginning in the early forties. There is therefore a need to provide more evidence-based practice standards to enhance health status, longevity, functional capability, and quality of life in this population[30].

A working group (Aging Special Interest Research Group) under the International Association for the Scientific Study of Intellectual Disabilities (IASSID) has recently published recommendations for further research areas[30]:

- The acquisition of additional clinical and epidemiological knowledge regarding specific syndromes with linkages to basic science research in biomolecular genetics and metabolism.
- The development of adapted diagnostic and therapeutic methods for people who have difficulties with cooperation or communication.
- The development and evaluation of interdisciplinary interventions for complicated conditions (like sensory impairment, dysphagia, communication, and functional decline).
- The development of clinimetric measures in a number of areas (functional capability, quality of life, mental health, pain assessment, and clinical diagnosis), that are sensitive and specific, easy to administer, and applicable to persons with a wide range of mental and physical capabilities.
- The evaluation of clinical guidelines, including referral protocols, to support community-based primary care physicians, within specific health care systems, to care for people with intellectual disabilities.
- The evaluation of the applicability of a new discipline of lifespan developmental medicine to lead in interdisciplinary care, health care education, service delivery, and research for people with intellectual disability.
- The development of the knowledge base regarding the health status and needs of people with intellectual disabilities living in less developed countries.

In the last few years, we here in Israel have embarked on several major studies of aging in the population of persons with ID and eventually we would like to follow the trend of our colleagues in Holland and establish a formalized subspecialty.

## BIOSKETCHES

**Joav Merrick, MD, DMSc,** is Professor of Child Health and Human Development affiliated with the Zusman Child Development Center and Division of Community Health at the Ben Gurion University, Beer-Sheva, Israel and presently the Medical Director of the Division for Mental Retardation, Ministry of Social Affairs, Jerusalem and the Director of the National Institute of Child Health and Human Development. He has numerous publications in the field of child and human development, rehabilitation, intellectual disability, disability, health, welfare, abuse, advocacy and prevention. Dr. Merrick received the Peter Sabroe Child Award for outstanding work on behalf of Danish Children in 1985 and the International LEGO-Prize ("The Children's Nobel Prize") for an extraordinary contribution towards improvement in child welfare and well being in 1987. E-mail: jmerrick@internet-zahav.net. Website: www.nichd-israel.com

**Isack Kandel**, **MA, PhD,** is Senior Lecturer at the Faculty of Social Sciences, Department of Behavioral Sciences, the Academic College of Judea and Samaria, Ariel. During the period 1985–93, he was Director of the Division for Mental Retardation, Ministry of Labour and Social Affairs, Jerusalem, Israel. E-mail: Kandeli@aquanet.co.il

**Mohammed Morad, MD**, is Specialist in Family Medicine, Lecturer in Family Medicine at the Division of Community Health, Ben Gurion University of the Negev and the Medical Director of a large area clinic in the city of Beer-Sheva. He has publications on Bedouin health, health aspects, spiritual health, and aging in persons with intellectual disability, and is a presenter on topics such as health policy and services for the disadvantaged at national and international conferences. E-mail: morad62@barak-online.net
